# Supplementary material for: Content-rich biological network constructed by mining PubMed abstracts
Source: BMC Bioinformatics. 2004 Oct 8;5:147. doi: 10.1186/1471-2105-5-147 (PMC528731; doi:10.1186/1471-2105-5-147)
Supplement: Additional File 2 — The original results of the above study (non-essential files are deleted to keep the file size under the limit set by BMC bioinformatics). [file 1471-2105-5-147-S2.bz2 › chilibotAdditionalFile2/dip05/541ID9891052E10894/html/PGR_UBE3A.html]

 


 **PGR** and **UBE3A** 
  
Found 3 abstracts in PubMed, retrieved 3.  
 

 What does Google say? 
 PDF only 
| .edu only 

---

**Interactive relationship** (e.g. stimulation, inhibition, etc)

**Non-interactive relationship** (e.g. studied together, co-existance, homology, etc.)

- In this study, we found that the E6 associated protein E6 AP  [ **UBE3A** ]   **UBE3A**  directly interacts with and coactivates the transcriptional activity of the human progesterone receptor  [ **PGR** ]  PR in a hormone dependent manner.  Ref: 9891052 Mol Cell Biol, 1999
